# An exceptional complication of transesophageal ultrasound in a patient with Coronavirus disease

**DOI:** 10.1002/ccr3.4030

**Published:** 2021-03-04

**Authors:** Amine Bahloul, Rania Hammami, Aymem Bougharriou, Selma Charfeddine, Racha Smaoui, Leila Abid, Samir Kammoun

**Affiliations:** ^1^ Department of Cardiology Hedi Chaker Hospital Sfax Tunisia

**Keywords:** COVID‐19, echocardiography COVID, esophageal perforation, transesophageal ultrasound

## Abstract

EP after TEE represents a medico‐surgical emergency. Given the high rate of asymptomatic patients with COVID 19, the risk of contamination and the frailty of esophageal tissues, we should check coronavirus infection in every patient before TEE.

## Graphical abstract

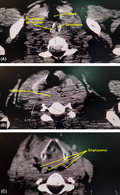

## 1 BACKGROUND

We present an unusual case of iatrogenic esophageal perforation (EP) following transesophageal echocardiography (TEE) in a patient with COVID‐19. The force applied during intubation on a fragile wall caused by COVID‐19 was likely the underlying condition contributing to the EP. Early diagnosis and urgent treatment are essential for a favorable prognosis.

Transesophageal echocardiography (TEE) is a very reliable method increasingly used in cardiology. TEE is nevertheless a semi‐invasive method which does have some risks. Esophageal perforation (EP) is a rare complication with an extremely low rate (0.02%‐0.09%)^1^, ^2^ but serious risk. We present the case of a patient with coronavirus disease who developed EP after TEE and we discuss if this complication is favored by COVID‐19 infection.

## 2 CASE PRESENTATION

Our patient was a 67‐year‐old woman with a medical history of hypertension and atrial fibrillation. She was admitted in our department because of one‐week fever (38.3°C). She had no respiratory symptoms or history of gastro‐esophageal disease. Physical examination revealed good general condition, with a blood pressure of 120/80 mm Hg, and a pulse rate of 80 beats per minute. Her oxygen saturation was 92% in ambient air. Pulmonary auscultation was normal. Cardiac auscultation showed normal heart sounds and a 3/6 systolic murmur to the mitral focus. The electrocardiogram was in sinus rhythm.

A transthoracic echography was performed, finding a slightly dilated left ventricle with preserved systolic function, severe mitral regurgitation by prolapse of the large mitral valve (A2 prolapse), and rupture of the chordae. There is moderate tricuspid regurgitation. Laboratory studies showed hemoglobin of 15.7 g/L and normal leukocyte count. There were elevated blood levels of C‐reactive protein (150 mg/L; normal range, 0‐10 mg/L).

**FIGURE 1:**
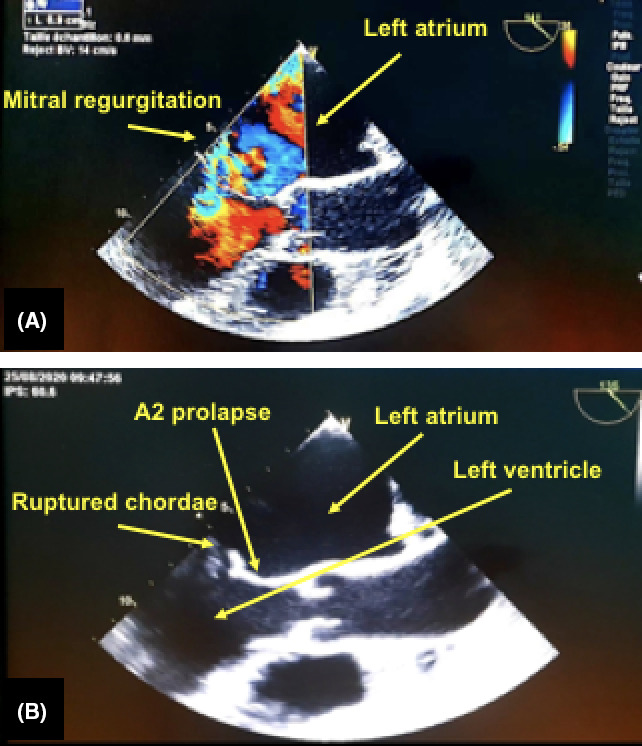
A transesophageal echocardiography showing a severe mitral regurgitation (A) by prolapse of the large mitral valve (A2 prolapse) and rupture of the chordae (B)

Given the high probability of infectious endocarditis (IE) suspected by the presence of fever, a biological inflammatory syndrome, and severe mitral regurgitation by chordae rupture, we performed TEE which did not show any signs of IE (Figure 1). The introduction of the probe was uneventful, and the patient tolerated well the examination.

**FIGURE 2:**
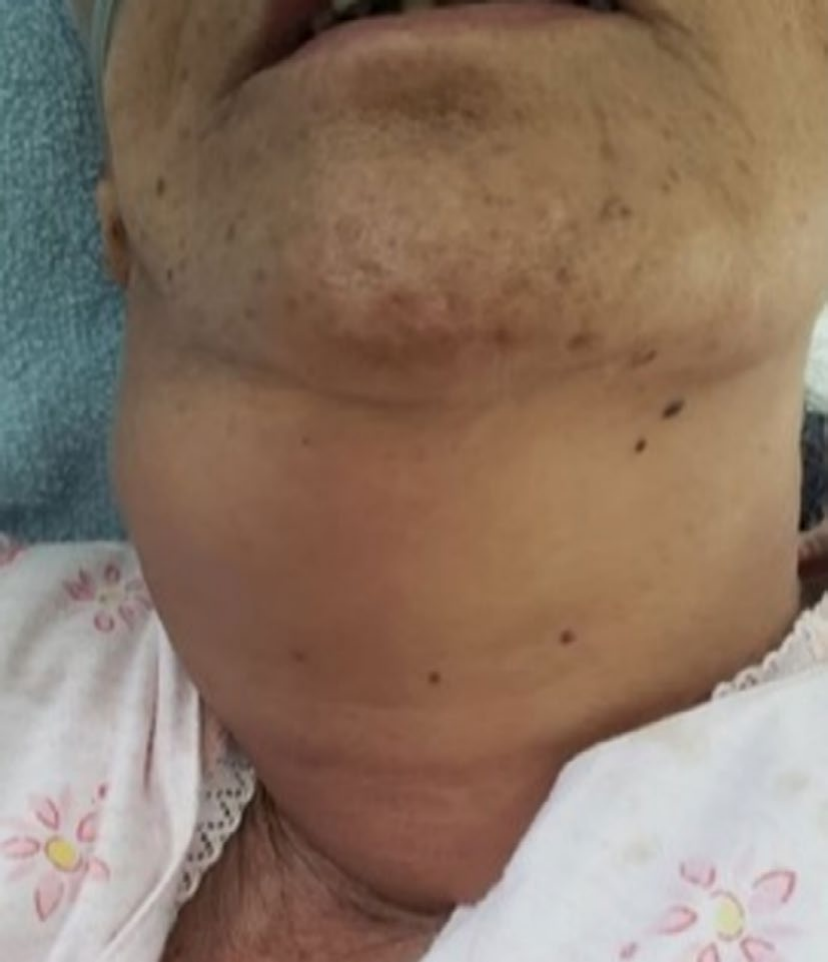
Cervical swelling related to hematoma and subcutaneous emphysema

Immediately after the TEE, the patient reported severe neck pain and cervical swelling. Examination of the cervical region revealed swelling of five centimeters in diameter consistent with hematoma and subcutaneous emphysema (Figure 2). The oropharyngeal examination was without abnormalities.

**FIGURE 3:**
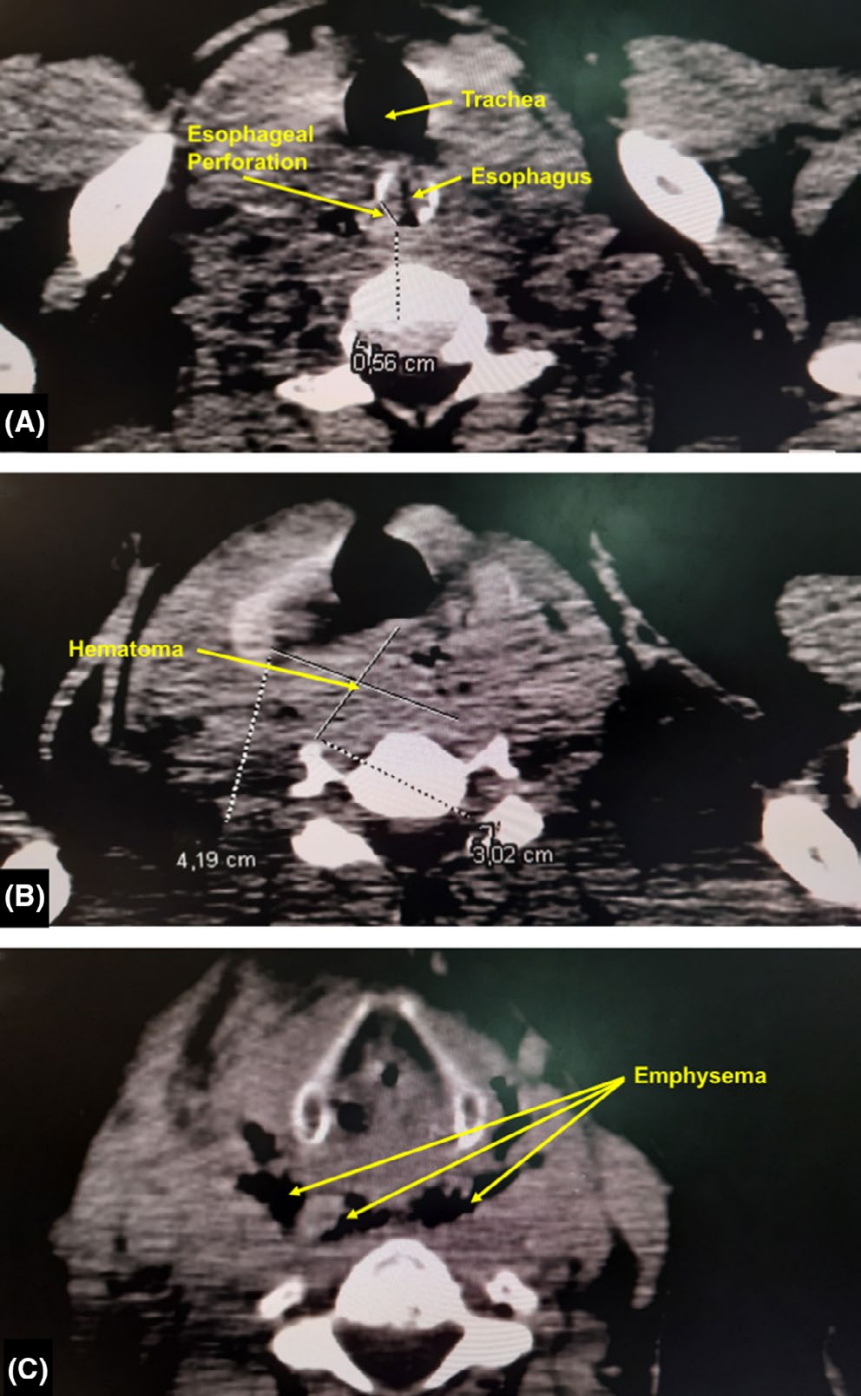
A cervicothoracic computed tomography scan after oral contrast administration showing a perforation (A) in the cervical esophagus, a hematoma (B) of the visceral space of the neck, and an emphysema (C) of the retropharyngeal space

A cervicothoracic computed tomography (CT) scan after oral contrast administration was performed showing perforation of the cervical esophagus, hematoma of the visceral space of the neck, and emphysema of the retropharyngeal space (Figure 3).

Intravenous antibiotics and parenteral nutrition were initiated. As a part of preoperative assessment, we performed a real‐time polymerase chain reaction (PCR) of nasopharyngeal swabs which was positive for SARS‐CoV‐2. The patient underwent emergent surgery, allowing the closure of the perforation, drainage of the collection, and feeding jejunostomy. Intraoperatively, the esophageal wall was inflammatory with evidence of reduced visceral perfusion throughout the esophagus. Unfortunately, our patient died, ten days later from severe refractory hypoxemia due to acute respiratory distress syndrome.

## 3 DISCUSSION

Here, we reported the first case of EP in our department during a TEE examination; more than 6000 examinations have been performed until now, the fact that the patient had COVID‐19 appears to have increased the risk of this complication. We considered that the perforation could be related to SARS‐CoV2 infection due to positive PCR test, the presence of a biological inflammatory syndrome on admission with elevated blood levels for C‐reactive protein, the inflammation of the esophageal wall observed intraoperatively, and the severe form of COVID infection which led to the patient's death. Unfortunately, we did not perform a histopathological examination of the esophageal wall.

EP is a rare condition, and the incidence is estimated at 3 cases per million inhabitants per year,^3^ but it remains a serious complication, associated with high mortality, mainly due to septic complications such as mediastinitis.^4^ The main causes are increased intraesophageal pressure (spontaneous or Boerhaave syndrome), iatrogenic damage due to instrumentation, physical or chemical trauma, or diseases of the esophagus.^3^

EP after TEE is extremely rare and commonly occurs in the cervical esophagus.^1^, ^2^, ^5^, ^6^ Although the incidence of perforation is low, esophageal mucosal injuries during TEE are common (up to 60%).^7^ However, only 2% of those injuries are recognized clinically. A review of the literature found 35 reported cases of EP secondary to a TEE.^8^ These were most often elderly women and TEE performed intraoperatively, without any particular difficulty during the procedure. EP during intraoperative TEE is mainly caused by direct trauma related to probe introduction and manipulation. Prolonged, continuous pressure and thermal energy from a probe can also damage esophageal tissue, resulting in indirect mechanical trauma.^9^

At the cervical level, the esophageal wall showed weakness caused by the crossing of fibers from the constrictor of the pharynx muscle and the cricopharyngeal muscle.^7^ This zone projects at the cervicothoracic junction (C5‐C6 vertebrae). Perforation risk at this level is increased during passing of the probe by upper extension of the neck. Flexion of the neck enables opening the cervicothoracic junction and decreases the risk of perforation.^7^

Identification of risk factors and gentle probe manipulation may prevent this complication.^10^, ^11^ Risk factors for perforation when instrumenting the esophagus appear to be spasm or hypertrophy of the cricopharyngeal sphincter and intrinsic esophageal disease (eg, inflammation). In these instances, increased mucosal friability and decreased esophageal compliance may increase the risk of perforation when passing the probe. The inflammation and ischemia of the esophageal wall reported here may result from septic and thromboembolic phenomena, caused directly or indirectly by the viral infection.^12^, ^13^ Additionally, the coronavirus has an extensive tissue distribution, causing microthrombosis and generalized small vessel vasculitis.^13^, ^14^ Many observational studies have stated that COVID‐19 causes a hypercoagulable state, with manifestations of intravascular coagulation.^13^, ^15^, ^16^ These phenomena, associated with patient comorbidities, such as hypertension and heart disease, are associated with high mortality rates. In our patient, EP may have been caused by direct damage of the esophagus wall by the extremity of the probe into an esophageal mucosal disturbance.

The presenting clinical signs are variable, including pain, hypotension, shock, fever, dyspnea, pneumomediastinum, and biological inflammatory syndrome.^8^, ^17^, ^18^ Subcutaneous emphysema confirms the diagnosis, but it is quite common with perforation of the cervical esophagus (60% of cases).^19^

Cervicothoracic CT scan after oral contrast administration is the gold standard investigation to confirm the diagnosis by detecting the presence of air in the mediastinum and visualizing of the perforation.^20^ It makes it possible to assess the perforation to guide treatment by determining the site of the perforation, its extent, the presence of abscesses, collections, and pleural effusions.

When minor rupture is evident, medical therapy consisting of board‐spectrum antibiotics and total parenteral nutrition may be attempted. Endoscopic treatment is also possible for small uncomplicated perforations diagnosed early. In more extensive cases, surgical treatment is performed, justified by the severe prognosis of this affection. Jones and Ginsberg^18^ reported mortality rates of 6%, 34%, and 29%, respectively, for cervical, thoracic, and abdominal esophageal perforation in a collected review. Surgical technique depends on the location of the perforation, its size, the viability of the esophageal wall, the extent of local sepsis, and the presence of an underlying esophageal lesion.^21^ Conservative surgical treatment involves debriding infected and necrotic tissue, suturing the puncture, and draining on contact. A feeding jejunostomy is placed at the same time.

## 4 CONCLUSION

In conclusion, we presented an unusual case of iatrogenic esophageal perforation in a patient with COVID‐19. Force applied during the intubation into an esophageal mucosal disturbance cause by COVID‐19 probably represented the underlying condition that contributed to the damage caused by the probe. We should avoid TEE in patients with COVID‐19, not only because of the risk of contamination but also an eventual frail esophagus and a high risk of perforation.

## CONFLICT OF INTEREST

None of the authors report a conflict of interest.

## AUTHOR CONTRIBUTIONS

ABA, RS, and ABO: managed the patient. ABA, RH, SK, LA, and SC: performed the analysis. ABA: wrote the manuscript. All authors reviewed and approved the final version of the manuscript.

## ETHICAL APPROVAL

Written informed consent was obtained from the patient's parents for publication of this case report and any accompanying images. All the procedures performed in this study were in accordance with the ethical standards of the institutional and national research committee.

## Data Availability

The data that support the findings of this study are available from the corresponding author upon reasonable request.
